# Massive Fecaloma Dissolved by Coca-Cola: A Case Report

**DOI:** 10.7759/cureus.107453

**Published:** 2026-04-21

**Authors:** Victoria M Estevez, Salar Javanshir, Jillian Lane, Daniel Shlyak, Mohammed M Masri

**Affiliations:** 1 Surgery, Lake Erie College of Osteopathic Medicine (LECOM) - Bradenton, Bradenton, USA; 2 Surgery, University of Florida, Gainesville, USA; 3 Trauma Surgery, St. George's University School of Medicine, True Blue, GRD; 4 Anesthesiology and General Surgery, Larkin Community Hospital, South Miami, USA; 5 General Surgery, Larkin Community Hospital, South Miami, USA

**Keywords:** case report, coca-cola, constipation, fecaloma, stercoral colitis

## Abstract

Fecalomas are hardened masses of fecal material that are a rare complication of chronic constipation and require surgical resection when conservative measures fail. Recent reports suggest that Coca-Cola, traditionally used to dissolve gastric phytobezoars, may also aid in the dissolution of fecalomas. We report the case of an 82-year-old woman with dementia, type 2 diabetes, hypertension, hypothyroidism, chronic gastritis, and depression who presented with acute abdominal pain. She had not produced stool or passed gas per rectum for several days prior to admission. Physical examination revealed middle and lower abdominal tenderness and distension. Computed tomography (CT) scan of the abdomen and pelvis demonstrated a large fecaloma (maximum diameter 12.2 cm) in the sigmoid colon and rectum, without evidence of perforation. Conservative management, including polyethylene glycol, senna, mineral oil, and sodium phosphate enemas, failed to resolve the fecaloma or the patient’s symptoms. Two 1 L Classic Coca-Cola enemas were then administered, resulting in a significant reduction in the size of the fecaloma on imaging and complete resolution of symptoms. With the return of bowel function, the patient avoided surgery and was able to be discharged on a maintenance bowel regimen. This case contributes to the increasing number of reports supporting Classic Coca-Cola as an adjunct to conservative therapies for fecalomas that are refractory to conservative management.

## Introduction

Chronic constipation, if not correctly managed, can cause fecal impaction, which is an acute accumulation of hardened stool. Fecal impaction itself can progress to long-standing, hardening masses of the stool, also known as fecalomas [1]. Fecalomas are most commonly found in the sigmoid colon and rectum, although rare cases have been reported in the jejunum and other bowel segments [1]. The prevalence of fecal impaction increases with age, rising from 3% in patients in their third decade of life to 16% in patients over 70, with a median age of 64 [2]. Risk factors include chronic constipation and neurological disorders, such as Parkinson’s disease and Chagas disease, as well as other conditions associated with impaired gastrointestinal motility [3]. Clinical manifestations include abdominal pain, distension, urinary retention, and, in severe cases, megacolon or bowel obstruction. If left untreated, complications such as stercoral colitis, bowel necrosis, and perforation can occur [4].

Management ranges from conservative measures (oral and rectal laxatives, enemas, manual disimpaction) to minimally invasive endoscopic treatments and surgery in refractory or complicated cases [5,6]. Traditional conservative approaches are not always effective, with failure rates reported at approximately 28.9% of patients, prompting interest in exploring non-invasive alternative therapies [7]. Given the morbidity associated with surgical intervention, interest remains in identifying safe, non-invasive alternatives for refractory cases.

One unconventional but potentially useful approach is the administration of Classic Coca-Cola as an enema, extrapolated from its established role in dissolving gastric phytobezoars [8]. Classic Coca-Cola acts as a mucolytic agent, a source of carbonic acid, and an effervescent solvent [9,10]. However, the environment of the colon differs substantially from that of the stomach in terms of luminal pH, motility patterns, and mucosal exposure. Unlike the acidic gastric milieu, the colon maintains a near-neutral pH. Despite these anatomical and physiological differences, isolated case reports have described the use of Coca-Cola enemas for fecaloma dissolution with favorable outcomes [11,12].

This case adds to the limited literature by detailing the clinical and procedural use of a Classic Coca-Cola enema for refractory fecaloma and examining its role in current management.

## Case presentation

An 82-year-old woman with type 2 diabetes, hypertension, hypothyroidism, chronic gastritis, and major depressive disorder presented with two days of abdominal pain, nausea, and vomiting. She had multiple risk factors for fecaloma formation, including chronic constipation, dementia, and reduced mobility, as she was non-ambulatory. Medication reconciliation revealed no use of opioids or anticholinergic agents.

On presentation, the patient was afebrile and hemodynamically stable. The abdomen was distended, dull to percussion, and diffusely tender without rigidity, guarding, or rebound tenderness. Neurologically, she was alert but a poor historian due to dementia.

Laboratory evaluation was notable for chronic anemia (hemoglobin range: 7.1-8.4 g/dL) and fluctuating hypokalemia (range: 3.2-3.8 mEq/L) during the hospitalization. 

**Figure 1:**
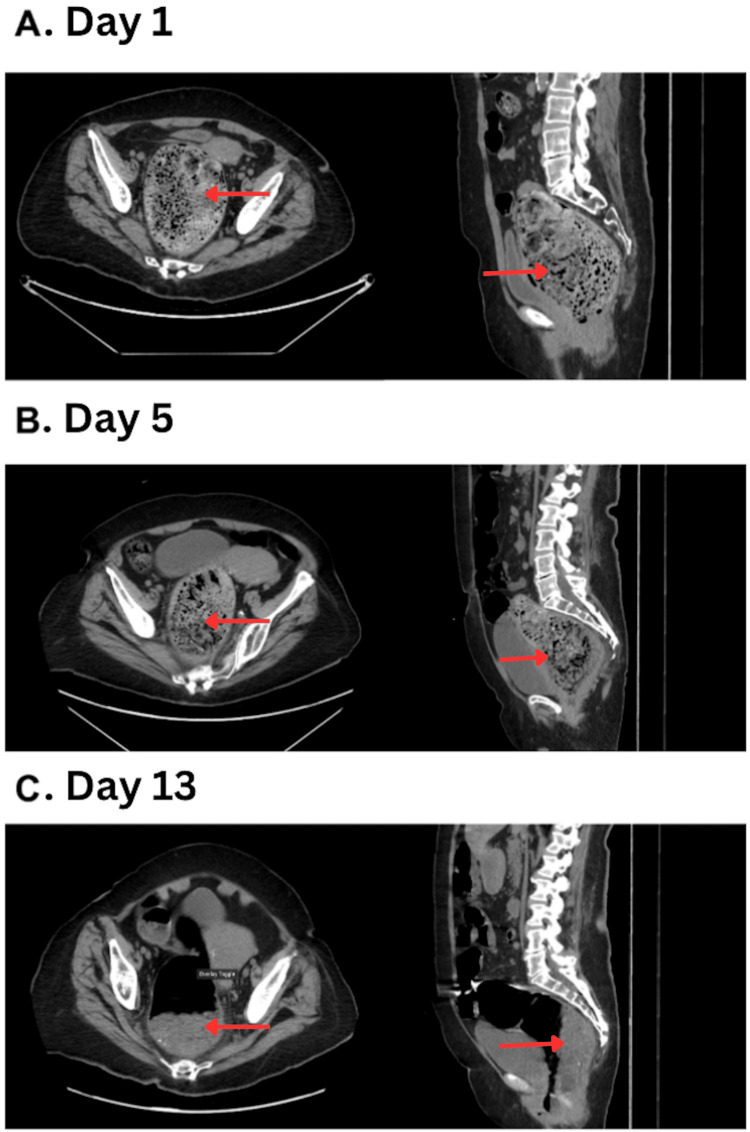
CT imaging of the patient during hospitalization. Panel A demonstrates the significant fecal impaction on admission. Panel B indicates fecaloma persistence after conservative measures on hospital day 5. Panel C shows significant reduction in size of the fecaloma one day after administration of a classic Coca-Cola enema. The red arrows indicate stool burden over the hospital course.

Computed tomography (CT) of the abdomen and pelvis without contrast revealed a large fecaloma, measuring 12.2 × 10.5 centimeters (cm), impacted in the sigmoid colon and rectum without surrounding inflammation or perforation (Figure 1A). Manual disimpaction was attempted on admission but was unsuccessful due to the inability to reach the fecaloma. Conservative management with senna, mineral oil, and polyethylene glycol 17 grams twice a day, and a sodium phosphate enema was attempted. Despite conservative treatment, symptoms of abdominal pain, distention, nausea, and vomiting persisted over the next four days. Repeat CT on hospital day 5 showed no significant change in fecaloma size (Figure 1B), and conservative therapy was continued without symptom improvement. 

On hospital day 13, due to the lack of symptomatic relief and the patient’s poor functional status, alternative conservative treatments were considered. Ultimately, a 1000 milliliter (mL) Classic Coca-Cola enema was administered in standard fashion. The patient was placed in the left lateral decubitus position. The enema bag was prepared by clamping the hose near the enema bag, then filling the bag with 1000 mL of Classic Coca-Cola. The enema nozzle was lubricated and gently inserted into the rectum to a depth of about 10 cm. The hose was unclamped, and the enema was allowed to flow gently under gravity. Care was taken never to force the enema into the patient. The patient was placed back into the supine position, and the enema bag was raised as high as the tubing would allow. After approximately 10 minutes, the entirety of the Classic Coca-Cola had been instilled. The hose was then clamped, and the enema was allowed to dwell for 60 minutes. Finally, the enema hose was unclamped, and feculent material was allowed to drain for 30 minutes. The nozzle was removed, completing the procedure.

The patient was able to tolerate the entire 1000 mL without discomfort. By the following day, the patient’s abdominal pain and vomiting had fully resolved, although abdominal distention persisted. A CT of the abdomen and pelvis demonstrated a 50% reduction in fecaloma size (Figure 1C). During this period, the patient was routinely monitored for changes in abdominal pain, bowel movements, hemodynamics, and daily blood work was obtained to monitor for electrolyte disturbances. A second 1000 mL Classic Coca-Cola enema was administered on hospital day 16, resulting in complete symptom resolution. The patient was discharged the following day with a maintenance bowel regimen including oral polyethylene glycol 17 grams twice daily and senna 8.6 milligrams once daily. Attempts to follow up with the patient were made, but she was lost to follow-up.

## Discussion

Fecalomas are uncommon but significant complications of chronic constipation, most often reported in elderly women with chronic constipation, neurogenic bowel dysfunction, or associated with Parkinson’s disease [6,11]. Complications include bowel obstruction, stercoral ulceration or perforation, and urinary retention [4,13]. Standard treatment involves stepwise escalation from laxatives and enemas to endoscopic fragmentation or surgical resection of the affected colon [6,11].

The novel use of Classic Coca-Cola enemas in fecaloma management stems from its success in dissolving phytobezoars [14]. Its acidity (pH ~2.5), sodium bicarbonate content, and CO₂ release facilitate softening and fragmentation. In phytobezoars, Coca-Cola achieves a considerable success rate as monotherapy or in combination with endoscopy [14]. 

Less than a dozen case reports describe Classic Coca-Cola’s use in fecalomas. Ontanilla et al. resolved a sigmoid fecaloma with Classic Coca-Cola irrigation [12]. Kang and Lim reported successful cola injection for a cecal fecaloma after endoscopic fragmentation with alligator grasping forceps, a tripod basket, and a net failed due to the fecaloma’s size and slick outer surface [9,11]. Lee and Kim demonstrated rapid fragmentation of a sigmoid fecaloma within 10 minutes of Coca-Cola injection [9]. Attieh et al. used Coca-Cola and argon plasma coagulation to successfully dissolve a giant fecaloma in a 94-year-old male with Parkinson’s disease [15]. In another study, Seth successfully used colonoscopic Coca-Cola administration to dissolve fecalomas in two separate instances [16]. These reports note the rapid symptomatic relief after standard conservative treatments failed and warrant further investigation. While we cannot draw a causal association, this case report can be a step toward proof of concept.

Our case aligns with these findings, suggesting Coca-Cola may be considered as an adjunct in refractory cases before endoscopic fragmentation or surgical intervention. Advantages include wide availability, low cost, and minimally invasive administration. Limitations include the small number of published cases (<10), all of which are uncontrolled and anecdotal in nature, precluding meaningful assessment of efficacy or reproducibility. There are no standardized protocols regarding concentration, volume, dwell time, or frequency of administration, and long-term safety data are lacking. In addition, structured monitoring parameters have not been defined. Although adverse events such as mucosal injury, electrolyte disturbances, or systemic effects, including systemic caffeine absorption, have not been reported in the existing literature, these risks remain theoretical concerns and warrant careful consideration. Collectively, these limitations prevent the establishment of a robust scientific foundation to support the routine clinical recommendation of Classic Coca-Cola enemas for fecaloma management. Prospective pilot studies or small cohort investigations are needed to better define safety, feasibility, and therapeutic benefit.

## Conclusions

Fecalomas are rare but clinically significant complications of chronic constipation that can lead to severe morbidity and mortality. Invasive endoscopic or surgical treatments are required for refractory cases that fail first-line treatments with oral laxatives. Limited evidence from fewer than a dozen case reports suggests that Classic Coca-Cola, in the form of an enema, may facilitate rapid softening and fragmentation of fecalomas, avoiding invasive therapies. Our case adds to the small body of literature available and supports Classic Coca-Cola enemas as a potential adjunct before endoscopy or surgery is required. However, the evidence remains anecdotal, with no standardized protocols, controlled studies, or long-term safety data. Further prospective studies are needed to better define the safety and effectiveness of Classic Coca-Cola enemas prior to recommending its widespread use.
